# Should We Treat SIBO Patients? Impact on Quality of Life and Response to Comprehensive Treatment: A Real-World Clinical Practice Study

**DOI:** 10.3390/nu17071251

**Published:** 2025-04-03

**Authors:** Ana Reyes Liébana-Castillo, Lucía Redondo-Cuevas, Ángela Nicolás, Vanessa Martín-Carbonell, Laura Sanchis, Aroa Olivares, Francisco Grau, Marina Ynfante, Michel Colmenares, María Leonor Molina, José Ramón Lorente, Héctor Tomás, Nadia Moreno, Ana Garayoa, Mercedes Jaén, María Mora, José Gonzalvo, José Ramón Molés, Samuel Díaz, Noelia Sancho, Eva Sánchez, Juan Ortiz, Vicente Gil-Guillén, Ernesto Cortés-Castell, Xavier Cortés-Rizo

**Affiliations:** 1 Valencian Digestive Institute (IVADI), 46021 Valencia, Spain; anareyesliebana@gmail.com (A.R.L.-C.); angela.nicolas@gmail.com (Á.N.); vanessamartin7891@gmail.com (V.M.-C.); laurasanchisartero@gmail.com (L.S.); aroa@clinicafimed.com (A.O.); ynfafer@hotmail.com (M.Y.); michelcobul6@gmail.com (M.C.); mlmolina293@gmail.com (M.L.M.); joselorentemontoro@gmail.com (J.R.L.); htomas@comv.es (H.T.); nadiapa88@hotmail.com (N.M.); mercedes.jaen.revuelta@gmail.com (M.J.); mariamoraescrig@gmail.com (M.M.); jrmolesmarco@gmail.com (J.R.M.); samueldiaznutricion@gmail.com (S.D.); noeliasanchopsic@gmail.com (N.S.); evasramos.91@gmail.com (E.S.); juanortizsalvador@gmx.com (J.O.); xacori@gmail.com (X.C.-R.); 2 Digestive Section, Hospital de Sagunto Internal Medicine Service, 46520 Valencia, Spain; anagarayoa83@gmail.com (A.G.); dianocito@gmail.com (J.G.); 3 Department of Pharmacology, Pediatrics and Organic Chemistry, Miguel Hernández University of Elche, 03550 Elche, Spain; vgil@umh.es (V.G.-G.); ernesto.cortes@umh.es (E.C.-C.)

**Keywords:** Small Intestine Bacterial Overgrowth (SIBO), quality of life, gut microbiota, herbal therapies, antibiotics, probiotics, FODMAP diet

## Abstract

**Background**: Small intestinal bacterial overgrowth (SIBO) is a dysbiosis marked by an excessive proliferation of bacteria in the small intestine, resulting in abdominal symptoms that significantly affect patients’ quality of life. **Objectives**: This study aims to evaluate the impact of a comprehensive therapeutic approach in improving the quality of life of patients with SIBO. **Methods**: For this purpose, standardized questionnaires were used at baseline, 30 days and 90 days, including the IBS-QOL (Irritable Bowel Syndrome Quality of Life Questionnaire), the GSRS (Gastrointestinal Symptom Rating Scale), the EuroQOL-5D, and the Bristol Scale. **Results**: The results show that a comprehensive approach, combining pharmacological treatment, appropriate dietary intervention, and strategies aimed at improving gut microbiota and intestinal permeability, produces a sustained improvement in the quality of life of a significant proportion of patients who participated in the study. Furthermore, the results suggest that, although gas normalization is a relevant indicator, clinical improvement and quality of life depend considerably on patients’ subjective perception of their health. **Conclusions**: This finding underscores the importance of recognizing SIBO as a prevalent condition that requires accurate diagnoses and individualized treatments to improve patients’ well-being.

## 1. Introduction

The microbiota, comprising various microbes symbiotically interacting with the host, plays a crucial role in numerous bodily functions, including digestion, defense mechanisms, metabolism, and endocrine regulation. This intricate balance, known as eubiosis, can be disrupted by various factors, leading to dysbiosis, characterized by an imbalance in the microbiota composition [1,2]. Small intestinal bacterial overgrowth (SIBO) is one consequence of dysbiosis in the small intestine, prevalent among patients with irritable bowel syndrome (IBS) [3]. With symptoms such as abdominal bloating, flatulence, and altered bowel habits, SIBO often extends beyond the digestive system, contributing to extraintestinal complications [2,4].

SIBO encompasses various types, primarily classified based on the predominant gas excreted by the microorganisms: hydrogen (H_2_-SIBO), methane (CH_4_-SIBO), and hydrogen sulfide. Notably, patients may present with a combination of gases, complicating diagnosis and management [5].

Diagnostic criteria for SIBO have evolved from invasive jejunal aspirate cultures to non-invasive breath tests, enabling earlier detection and a surge in estimated incidence rates [6]. Treatment typically involves antibiotics, yet their efficacy varies, prompting exploration of alternative therapies [7].

Herbal supplements have emerged as promising adjuncts to antibiotic therapy, with studies demonstrating comparable effectiveness in SIBO management [8,9]. Additionally, probiotics, glutamine, and prebiotic fiber have shown potential in alleviating SIBO symptoms and improving patient outcomes [10,11,12]. Regardless of advancements, challenges persist, including the lack of consensus on dietary strategies and high relapse rates post-antibiotic treatment [13]. Addressing these gaps necessitates a holistic approach focusing on modifying the bacterial ecosystem to enhance dysbiosis resolution and patient well-being.

Despite the increasing recognition of small intestinal bacterial overgrowth (SIBO) within the medical community and the availability of guidelines from major digestive scientific societies regarding its management, many healthcare professionals still fail to provide adequate care and treatment for affected patients. This skepticism toward the condition may lead to underdiagnosis and, consequently, suboptimal management, ultimately compromising patients’ quality of life [7].

There remains significant uncertainty regarding the optimal management of SIBO to improve clinical response and breath test outcomes. Moreover, there is even less evidence regarding the health status and quality of life of these patients and whether disease management enhances their quality of life. It is imperative to recognize the substantial impact of SIBO on patients’ quality of life, akin to that observed in patients with IBS. This assertion is supported by findings from Casellas Jordà and López Vivancos [14], whose study corroborated that IBS has a significant impact on numerous aspects of life, not only due to the impact of symptoms but also due to functional repercussions. However, unlike IBS, the evaluation of such impacts in SIBO remains underexplored, particularly regarding clinical responses to these aspects of quality of life post-treatment.

In summary, as the landscape of SIBO management continues to evolve, there is a critical need for comprehensive evaluation strategies that encompass not only clinical outcomes but also patient-reported outcomes related to quality of life and overall well-being.

Therefore, our hypothesis is that the comprehensive management of SIBO, integrating clinical support from physicians alongside dietary guidance from a nutritionist focusing on the low FODMAP (fermentable oligo-, di-, and monosaccharides and polyols) diet, supplemented with probiotics, glutamine, and prebiotic fiber, may serve as a significant clinical tool for improving or normalizing the quality of life of patients with SIBO.

## 2. Materials and Methods

### 2.1. Study Population

A total of 179 patients diagnosed with SIBO were enrolled based on the measurement of hydrogen and methane gases in exhaled breath. These individuals visited the Gastroenterology Department of Sagunto Hospital and Casa de Salud Hospital in Valencia, Spain between November 2021 and March 2023.

The inclusion criteria involved a confirmed diagnosis of hydrogen or methane SIBO, compliance with the prescribed treatment for three months, and the signing of an informed consent. Exclusion criteria consisted of being younger than 18, having a related chronic digestive disorder (such as celiac disease or inflammatory bowel disease), the presence of intestinal parasites, elevated fecal calprotectin levels, or any other potential cause of dysbiosis that might influence treatment outcomes, per assessment by a gastroenterologist.

### 2.2. Data Collection

Data were gathered between November 2021 and March 2023, and all patients were monitored from their initial diagnosis throughout the subsequent 3 months of treatment.

#### 2.2.1. SIBO Diagnosis

Each patient underwent a breath test at the start and after 3 months of study. In this procedure, the patient consumes a substrate (either lactulose or lactitol) and exhaled breath samples are collected every 25 min over a 175-minute period. A graph displaying the concentrations of hydrogen, methane, and carbon dioxide (CO_2_) was generated from the results. CO_2_ levels were assessed to confirm proper sample collection. If hydrogen levels increased by 20 ppm from baseline prior to the 90th minute, the result was deemed positive for H_2_-SIBO. If methane levels exceeded 10 ppm at any point on the graph, the result was considered positive for CH_4_-SIBO.

#### 2.2.2. Complementary Analytical Tests

A routine blood test was conducted at the beginning and end of the treatment to assess the patient’s general health status, alongside stool cultures, fecal calprotectin, and fecal parasite tests. The analysis also included tests for B12, D vitamins, folic acid, anti-transglutaminase antibodies, hemogram, liver enzymes, iron metabolites, and C-reactive protein.

#### 2.2.3. Measurement of Covariates

In addition to age, sex, BMI, smoking habits, daily alcoholic intake, chronic diseases, and medication history, all patients were evaluated using standardized quality-of-life questionnaires. These included the Irritable Bowel Syndrome Quality of Life (IBS-QOL) questionnaire, the Gastrointestinal Symptom Rating Scale (GSRS), and the EuroQOL-5D questionnaire. In addition to the aforementioned questionnaires, patients were asked to indicate the type of stool they had during the last 4 weeks using the Bristol scale.

The IBS-QOL questionnaire is a tool designed to assess the quality of life in individuals with irritable bowel syndrome (IBS) [15]. The adapted and validated Spanish version of the IBS-QoL [16] includes 34 items presented as descriptive statements and covers eight subscales: emotional, mental, sleep pattern, energy, physical activity, dietary adaptations, sexual life, and social and work environment. Each item is rated on a 5-point scale to measure how well the description aligns with the respondent’s feelings: not at all, slightly, moderately, somewhat, and extremely or a great deal. All responses are summed and standardized (0–100) to compute the total score and subscale scores, where lower scores reflect better health-related quality of life (HRQoL). This tool evaluates how IBS impacts various aspects of the patient’s life. Higher IBS-QOL scores signify a better quality of life in relation to IBS.

Additionally, the Spanish version of the EuroQoL-5D was administered [17,18]. The EuroQoL-5D is a standardized tool for evaluating health-related quality of life (HRQoL). It consists of two main components: a descriptive system that evaluates five health dimensions (mobility, self-care, usual activities, pain/discomfort, and anxiety/depression), and a visual analog scale (VAS) that measures overall health status. In the descriptive system, the first component of the EuroQoL-5D, patients rate their health status on the day of the interview across the five dimensions mentioned. The second component involves the VAS, where respondents assess their overall health on a 20-cm vertical scale, marked with anchors at 0 (indicating the worst possible health status) and 100 (representing the best possible health status), often referred to as the “feeling thermometer.”

The GSRS is a questionnaire designed to evaluate gastrointestinal symptoms in patients with various conditions, including IBS [19,20], and contains 15 items that assess the severity of GI symptoms over the past seven days. The items address the severity of symptoms such as abdominal pain, pain relieved by a bowel movement, bloating, flatulence, constipation, diarrhea, loose stools, hard stools, urgent need to defecate, incomplete bowel emptying, fullness shortly after eating, fullness long after meals, and visible abdominal distension. Each item is rated on a 1–7 scale, where 1 means “no discomfort” and 7 means “extremely severe discomfort” related to the symptom. Higher scores on the GSRS indicate more severe gastrointestinal symptoms.

The Bristol scale [21] is a useful tool for classifying stool according to its shape and consistency. This classification into seven types can help to better understand digestive health and detect possible digestive motility conditions. Types 1 and 2 indicate constipation, types 3 and 4 are considered normal, and types 5, 6, and 7 are associated with diarrhea.

Participants completed the three questionnaires (IBS-QOL, EQ-5D, and GSRS) and the Bristol scale under the supervision of trained medical staff. Understanding of the instructions was ensured, and assistance was provided in case of any doubts. Questionnaire administration took place in hospital to ensure appropriate conditions for completion. Each questionnaire was administered at the beginning of the study, at 4 weeks, and at 3 months follow-up to evaluate changes in symptom severity over time.

#### 2.2.4. Procedure

To be included in the study, patients had an initial consultation with a gastroenterologist and gave their consent by signing the informed consent form. During this visit, the results of the breath test were recorded, confirming the diagnosis. A detailed medical history was obtained, covering anthropometric measurements (weight, height), smoking, alcohol consumption, and medication use, and a blood and stool analysis was also requested. Based on the type of SIBO, the physician prescribed the appropriate treatment. The patient then visited a dietitian–nutritionist, who recorded their dietary habits to recommend a personalized low FODMAP diet tailored to their needs and lifestyle, enhancing adherence and satisfaction with the diet. Antibiotic and medicinal herb treatments were initiated alongside the low FODMAP diet during the first month of treatment.

**Figure 1:**
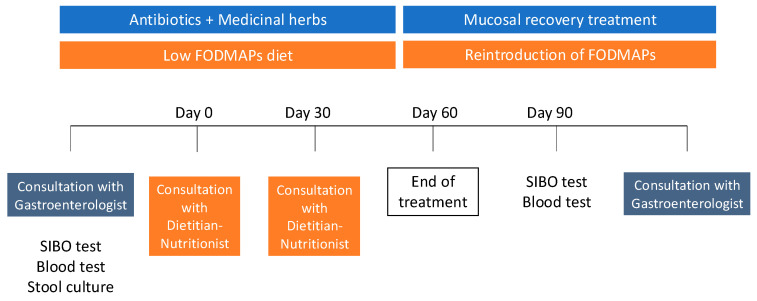
Diagram of the treatment time course (adapted from Redondo-Cuevas et al. [22]).

After 30 days of treatment, the patients returned for a follow-up consultation with the dietitian–nutritionist, who provided guidance on how to reintroduce foods during the following 8 weeks. At this stage, a new breath test for SIBO and another blood test were scheduled after 6 weeks of treatment (15 days prior to the final gastroenterologist consultation). After 90 days of treatment, patients had a final consultation with the gastroenterologist to review clinical symptoms and the results of both blood tests and the SIBO breath test (Figure 1) [22].

#### 2.2.5. Treatment

Treatment was comprehensive and integrated, focusing on dietary guidance from a nutritionist alongside clinical support from physicians. Treatment was tailored to each patient according to the type of SIBO. All patients received Rifaximin (along with Neomycin, if SIBO methane) plus a low FODMAP diet guided by a nutritionist. Herbs (oregano oil, peppermint, and berberine) were added to the GI (IVADI) followed by probiotics with L. rhamnosus CBI, Bifidum BB47, B. longum ES1, and L-Glutamine.

### 2.3. Statistical Analysis

Quantitative variables were presented as means and standard deviations, while qualitative data were reported using absolute (n) and relative (%) frequencies. Paired-sample Student’s *t*-test was used to identify potential differences between the initial and final values of the quantitative variables, while Chi-square tests were employed for qualitative variables. To compare the different SIBO subgroups, Student’s *t*-test was applied. Lastly, to control for potential confounding factors, binary logistic regression analysis was performed on all patients and within SIBO subgroups to assess the normalization of exhaled gases and clinical indicators after 3 months of treatment.

### 2.4. Ethical Considerations

The study adhered to international ethical guidelines for human research and clinical trials, as outlined in the Declaration of Helsinki, and received approval from the Clinical Research Ethics Committee of Sagunto Hospital (FXC-TNF-2015-01). All participants provided written informed consent.

## 3. Results

The characteristics of the analyzed patients are described in Table 1. Among the 179 patients with SIBO studied, 31.3% had hydrogen SIBO and 68.7% had methane SIBO. The mean age was 45.7 ± 16.2 years and 82.7% were women. After treatment, gas excretion normalized in 41.3% of patients (33.9% in hydrogen SIBO and 44.7% in methane SIBO). However, the clinical response was significantly better, with 72.6% of patients experiencing clinical improvement (71.4% in hydrogen SIBO and 73.2% in methane SIBO). No significant differences were observed between those who had normalized gas levels and those who did not in any of the analyzed variables (Table 1), both in gas normalization and clinical evolution, although a greater response was observed in CH_4_-SIBO. More details on these clinical results are provided in the 2024 published paper [22].

In Table 2, it can be observed that 100% of patients reported an improvement in their self-perceived health status, regardless of whether they achieved gas normalization or not. Notably, the difference between the initial and final values is greater among those who normalized gas levels in the post-treatment SIBO test. This difference is also observed between patients who normalized their clinical symptoms and those who did not.

In the GSRS scale (Table 3), a positive evolution is also observed. However, no significant differences are found between those who achieved gas normalization and those who did not, in both cases.

Similarly, an improvement in the IBS-QoL scale (Table 4) is observed throughout the treatment in all patients included in the study. However, once again, no significant differences are found between those who showed gas normalization and those with clinical symptom improvement. This trend is consistent across all analyzed domains (emotional, mental, sleep, energy, physical activity, diet, social, sexual, and occupational).

The evolution of stool quality was assessed using the Bristol Stool Scale at baseline, one month, and three months after treatment. Stool consistency was classified into three main categories: normal, hard (associated with constipation), and loose (associated with diarrhea).

**Figure 2:**
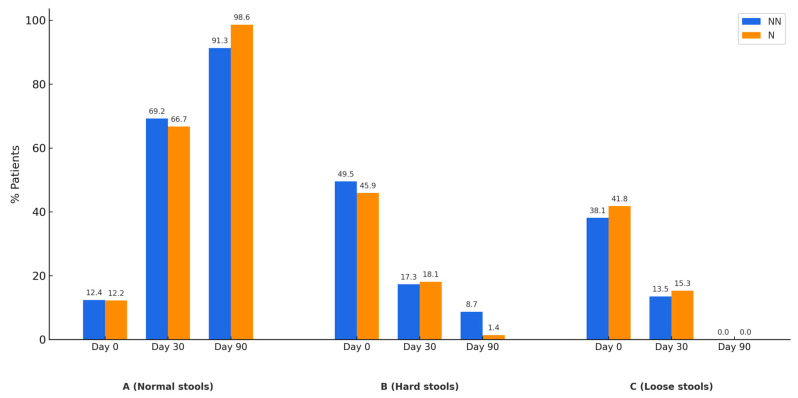
Evolution of the percentage of patients with normal stools (Panel **A**), hard stools (Panel **B**), or loose stools (Panel **C**) according to the Bristol Stool Scale. Patients who did not normalize gas levels are represented in blue (NN), while those who achieved normalization are shown in orange (N).

As shown in Figure 2A, the number of patients with normal stool consistency increases as treatment progresses. Conversely, the number of patients with hard stools (Figure 2B) and loose stools (Figure 2C) decreases at 30 and 90 days. These three patterns are observed in both patients who achieved gas level normalization after treatment and those who did not.

**Figure 3:**
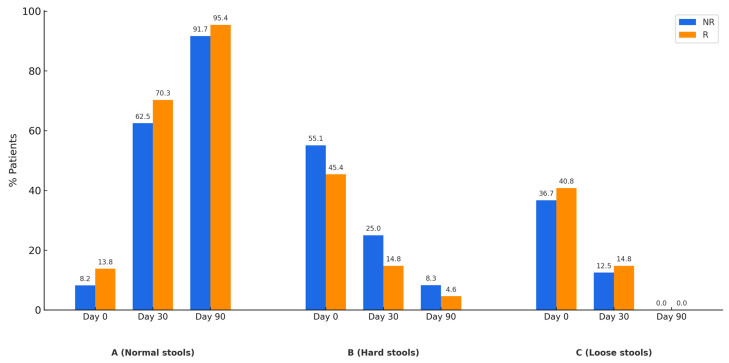
Evolution of the percentage of patients with normal stools (Panel **A**), hard stools (Panel **B**), or loose stools (Panel **C**) according to the Bristol Stool Scale. Patients who did not normalize clinical symptoms are represented in blue (NR), while those who achieved normalization are shown in orange (R).

In Figure 3A, it can be observed that as treatment progresses, the number of patients with normal stool consistency increases. Conversely, at 30 and 90 days, the number of patients with hard stools (Figure 3B) and loose stools (Figure 3C) decreases. These three patterns are observed in both patients who achieved clinical symptom normalization after treatment and those who did not.

Finally, to determine whether any initial factors might predispose patients to either gas excretion normalization or clinical symptom normalization, a logistic regression analysis was conducted. The analysis compared gas normalization (yes/no) against multiple variables (sex, age, SIBO type, and baseline scores from EQ-5D, GSRS, IBS-QoL, and Bristol Scale). No significant model was obtained when the analysis was performed for gas normalization (Chi-square = 8.365; *p* = 0.399).

However, when analyzing clinical symptom normalization, the model yielded a Chi-square = 10.009; *p* = 0.002, showing a significant association with the initial self-perception score on the EQ-5D visual scale (ExpB = 1.029; 95% CI 1.010–1.048; *p* = 0.002). The area under the ROC curve was 0.655 (95% CI 0.567–0.743; *p* = 0.001), indicating that a higher initial perception of well-being increased the likelihood of clinical symptom improvement.

## 4. Discussion

Despite the growing clinical and research attention on SIBO, there have been relatively few studies examining the quality of life in affected patients. To our knowledge, this is the first study with a large sample size that evaluates quality-of-life improvements in SIBO patients following treatment based on current international clinical guidelines [7,23].

Our results reveal that after treatment, 41.3% of patients achieved gas normalization, while 72.6% reported significant clinical improvement at the end of therapy [22]. This finding aligns with prior research suggesting that quality of life in SIBO or IBS patients is more strongly linked to clinical improvement rather than diagnostic test results [24]. Patients who achieved clinical symptom normalization showed significant improvements across quality-of-life scales (EQ-5D, GSRS, IBS-QoL), whereas those who only normalized gas levels without significant symptom reduction experienced less pronounced changes.

A general trend of improvement in self-perceived health was observed on the EQ-5D scale, with notable progress at 90 days. This highlights the importance of assessing and addressing clinical symptoms and subjective well-being, beyond merely focusing on objective breath test results for SIBO [25]. This is supported by recent guidelines suggesting that breath tests for the diagnosis of SIBO may be inaccurate and should be reconsidered in future diagnostic strategies [26].

The regression analysis indicated that initial self-perceived well-being on the EQ-5D visual scale was the most significant predictor of clinical improvement, reinforcing the relevance of individual perception in patient outcomes. This aligns with previous studies showing that a higher baseline self-perception of health increases the likelihood of clinical improvement in IBS and SIBO patients [27,28].

SIBO treatment has also been linked to mood improvements, reducing anxiety and depression, which are highly prevalent among these patients. This effect may be related to improved tryptophan metabolism, a key pathway in the gut–brain axis [29]. Furthermore, social support, stress, and anxiety have been identified as important factors affecting quality of life in IBS patients [30,31,32]. IBS patients with high stress and anxiety levels report poorer quality of life, suggesting that psychological interventions may be beneficial when combined with SIBO treatment [31,32].

These findings emphasize the importance of a multidisciplinary approach that considers both biological and psychosocial factors in managing SIBO and IBS. Future studies should explore the role of psychological interventions in improving the quality of life in SIBO patients, aiming for a more comprehensive and personalized treatment approach.

Regarding patient subgroups, those with methane-predominant SIBO (CH_4_-SIBO) showed a greater tendency toward clinical normalization. This supports previous research indicating that SIBO type influences treatment response and quality of life. CH_4_-SIBO, often associated with constipation symptoms, tends to impact quality of life differently compared to H_2_-SIBO, which is more commonly linked to diarrhea and other gastrointestinal symptoms [24,33]. Moreover, improvements in stool consistency (Figure 2), observed in both patients who normalized gas levels and those who did not, underscore the potential of treatment in regulating intestinal motility and stool quality. This is a critical aspect of quality of life for SIBO patients, as stool normalization may correlate with a greater sense of well-being and symptom control, consistent with findings in IBS research [34,35].

Additional studies on dietary effects, including low-FODMAP and gluten-free diets, have demonstrated quality-of-life benefits in IBS patients [36,37]. In our study [22], we implemented a low-FODMAP dietary intervention with reintroduction phases, which likely contributed significantly to the observed quality-of-life improvements. Previous research suggests that IBS patients who avoid or restrict specific foods tend to have more severe symptoms and lower quality of life [38]. This dietary strategy not only facilitates symptom control but may also optimize clinical responses, leading to gas normalization and symptom reduction, providing a more holistic and individualized treatment approach.

Finally, the use of herbal supplements and strategies to improve intestinal permeability in this study may have further enhanced patients’ quality-of-life perception. Many of the antimicrobial plants used [22], along with glutamine, prebiotic fibers, and probiotics, are also known to reduce visceral hypersensitivity [39,40,41,42,43]. In future studies, it would be very interesting to carry out an analysis of the gut microbiota, given that SIBO is related to a particular type of gut dysbiosis, in order to observe these improvements in gut permeability and gut microbiota composition.

A key finding of this study is that, with a substantial sample size, the majority of patients showed improvements in quality-of-life parameters by the end of the intervention. This highlights the effectiveness of our comprehensive approach, making it one of the most significant contributions of this research. Our results clearly indicate that SIBO-diagnosed patients, regardless of IBS or other comorbidities, experience notable mid-term improvements when receiving integrated treatment combining pharmacotherapy, supplementation, and diet. SIBO remains one of the most common conditions leading patients to consult primary care and gastroenterology specialists, yet many healthcare professionals remain skeptical about conducting diagnostic tests or prescribing targeted treatments for this large patient population [44,45,46]. Our real-world clinical practice data provide clear evidence that this comprehensive approach, incorporating dietary modifications and microbiota-targeted treatment, significantly improves patients’ quality of life in both the short and medium term. Consequently, generalizing this approach for SIBO management appears to be a reasonable course of action.

To date, and based on our knowledge and a comprehensive review of the available scientific literature, no study has specifically analyzed the quality of life in patients with small intestinal bacterial overgrowth (SIBO). Most available research addresses this topic indirectly or in combination with other conditions, such as IBS, which limits direct comparisons with our findings. In this context, our study makes a unique and valuable contribution as it is one of the few—if not the only—to specifically and thoroughly examine the impact of an intervention on quality of life in this patient population. The obtained results, which demonstrate a significant and consistent improvement across all patients, underscore the importance of this comprehensive approach and highlight the value of our study in an underexplored field.

Ultimately, further studies are needed to evaluate the long-term impact of different therapeutic approaches on the quality of life of SIBO patients, particularly considering the various strategies currently endorsed by international guidelines for SIBO management [47,48,49].

### Strengths and Limitations

One of the main limitations of this trial is the lack of a placebo or control group, which makes it difficult to separate the specific effects of the intervention from natural variability in symptoms or response to the placebo effect. This decision was made for ethical reasons, as it would not be appropriate to leave patients with significant symptoms untreated. Furthermore, as quality of life is a subjective measure, the observed improvement could be influenced by factors such as patient expectations or psychological effects related to the intervention. Randomized controlled clinical trials with larger and more diverse samples are needed to confirm these findings and determine the true magnitude of the effect of treatment. Additionally, we acknowledge the difficulty of isolating the effects of the different interventions in such a multimodal and comprehensive treatment. Our study seeks to evaluate the impact of a holistic approach, recognizing that the individual effects of each component cannot be completely separated.

Moreover, this study adds value by assessing not only symptom resolution but also the quality of life of SIBO patients, a key aspect that is often underestimated. Its medium- and long-term follow-up (30 and 90 days) allows the sustainability of the treatment to be analyzed and provides a more complete picture of its impact. In addition, the comprehensive approach, combining antibiotics, diet, and supplements, reflects standard clinical practice. The use of validated questionnaires and the inclusion of different subtypes of SIBO strengthen the validity of the study and will lay the foundation for future, more robust research.

## 5. Conclusions

The results of our study strongly suggest that a comprehensive approach combining pharmacological treatment, appropriate dietary intervention, and treatment aimed at improving gut microbiota and gut permeability leads to a substantial and sustained improvement in quality of life for a considerable percentage of SIBO patients in our study.

On the other hand, the results suggest that although gas normalization is a relevant indicator, clinical improvement and quality of life are highly dependent on patients’ subjective perception of their health status. This finding not only confirms the efficacy of our intervention but also reinforces the need to recognize SIBO as a highly prevalent clinical condition that requires accurate diagnosis and specific treatments to optimize patient well-being.

## Figures and Tables

**Table 1:** Sample description and comparison according to treatment success based on gas excretion curve and clinical criteria.

Variable	Total Sample (n = 179)	Gas Normalization n (%) 74 (41.3)	*p*-Value	Clinical Improvement n (%) 130 (72.6)	*p*-Value
Age Mean (years) ± SD	45.7 ± 16.2	43.8 ± 15.8	0.761	45.8 ± 16.9	0.413
Women n (%)	148 (82.7)	61 (82.4)	0.941	107 (82.3)	0.830
H_2_-SIBO n (%) CH_4_-SIBO n (%)	56 (31.3) 123 (68.7)	19 (33.9) 55 (44.7)	0.193	40 (71.4) 90 (73.2)	0.857

**Table 2:** Evolution of EQ-5D scale scores based on normalization of exhaled gases and clinical symptoms.

Time Evolution	Gas NN (n = 105) Mean (Score) ± SD	Gas N (n = 74) Mean (Score) ± SD	*p*-Value NN vs. N	Clinical NR (n = 49) Mean (Score) ± SD	Clinical R (n = 130) Mean (Score) ± SD	*p*-Value NR vs. R
Day 0	50.5 ± 20.9	54.5 ± 17.6	0.088	44.6 ± 18.2	55.0 ± 18.2	<0.001
Day 30	65.9 ± 18.0	67.5 ± 19.2	0.285	55.0 ± 17.9	70.9 ± 16.8	<0.001
Day 90	73.1 ± 21.1	81.0 ± 17.3	0.003	57.8 ± 21.2	83.4 ± 14.2	<0.001
*p*-Value i vs. f	<0.001	<0.001		<0.001	<0.001	

i at diagnosis (day 0); f after 3 months of treatment (day 90). NN, not normalized; N, normalized; NR, not resolved; R, resolved.

**Table 3:** Evolution of GSRS scale scores based on normalization of exhaled gases and clinical symptoms.

Time Evolution	Gas NN (n = 105) Mean (Score) ± SD	Gas N (n = 74) Mean (Score) ± SD	*p*-Value NN vs. N	Clinical NR (n = 49) Mean (Score) ± SD	Clinical R (n = 130) Mean (Score) ± SD	*p*-Value NR vs. R
Day 0	53.2 ± 6.5	52.5 ± 7.2	0.268	52.6 ± 5.8	53.0 ± 7.1	0.348
Day 30	23.0 ± 3.3	23.7 ± 7.1	0.235	22.7 ± 2.2	23.5 ± 6.0	0.196
Day 90	22.2 ± 4.1	22.5 ± 3.7	0.274	22.0 ± 3.0	22.4 ± 4.2	0.241
*p*-Value i vs. f	<0.001	<0.001		<0.001	<0.001	

i at diagnosis (day 0); f after 3 months of treatment (day 90). NN, not normalized; N, normalized; NR, not resolved; R, resolved.

**Table 4:** Evolution of IBS-QoL scale scores based on normalization of exhaled gases and clinical symptoms.

Time Evolution	Gas NN (n = 105) Mean (Score) ± SD	Gas N (n = 74) Mean (Score) ± SD	*p*-Value NN vs. N	Clinical NR (n = 49) Mean (Score) ± SD	Clinical R (n = 130) Mean (Score) ± SD	*p*-Value NR vs. R
Day 0	11.8 ± 1.2	11.6 ± 1.0	0.090	12.0 ± 1.0	11.6 ± 1.2	0.028
Day 30	13.7 ± 0.9	13.7 ± 1.0	0.338	11.6 ± 1.2	13.7 ± 1.0	0.325
Day 90	13.9 ± 1.2	13.9 ± 1.3	0.344	14.0 ± 1.1	13.9 ± 1.3	0.202
*p*-Value i vs. f	<0.001	<0.001		<0.001	<0.001	

i at diagnosis (day 0); f after 3 months of treatment (day 90). NN, not normalized; N, normalized; NR, not resolved; R, resolved.

## Data Availability

The original contributions presented in the study are included in the article, further inquiries can be directed to the corresponding author.
